# Laser Doppler mapping of lower limb skin perfusion before and 5 h after exercise in warm conditions

**DOI:** 10.3389/fphys.2026.1830186

**Published:** 2026-06-24

**Authors:** Jose I. Priego-Quesada, Emma Rebollar-Cuenca, Carlos Galindo-González, Carlos Sendra-Pérez, Alberto Hermo-Argibay, Rosa Ma Cibrián Ortiz de Anda

**Affiliations:** 1 Research Group in Sports Biomechanics (GIBD), Department of Physical Education and Sports, Universitat de València, Valencia, Spain; 2 Research Group in Medical Physics (GIFIME), Department of Physiology, University of Valencia, Valencia, Spain; 3 Department of Education and Specific Didactics, Jaume I University, Castellon, Spain; 4 Department of Endocrinology and Nutrition, University Hospital Doctor Peset, Foundation for the Promotion of Health and Biomedical Research in the Valencian Region (FISABIO), Valencia, Spain

**Keywords:** laser Doppler, running, skin blood flow, skin temperature, thermography

## Abstract

**Introduction:**

Although the use of laser Doppler perfusion imaging has a potential application to monitor internal load associated to exercise, its use in sport science is scarce and the relatively long scanning time required for each region of interest may constrain its use. The aim of this study was to evaluate the effect of running on skin perfusion five hours after exercise across different regions of interest in the lower limbs, and to examine the relationship between these perfusion responses and changes in other physiological and environmental outcomes.

**Methods:**

A total of 22 volunteers were divided into two groups: an exercise group, which performed a 50-min outdoor run between measurements, and a control group. Skin perfusion (laser Doppler perfusion imaging device, moorLDI2) and skin temperature (infrared thermography, Flir E54) in 6 regions of interest (anterior thigh, anterior knee, anterior leg, posterior thigh, posterior knee and posterior leg), heart rate and heart rate variability (Polar H10) and estimated core temperature (heat flux, Calera Research) were measured before and 5 hours after running in an outdoor temperature of 28 -31 °C.

**Results:**

The exercise group presented a higher increase of skin perfusion than the control group for the anterior thigh (95%CI[7, 58%], p=0.02 and ES = 1.0), anterior leg (95%CI[1, 79%], p=0.04 and ES = 0.3), and posterior thigh (95%CI[8, 56%], p<0.01 and ES = 0.5). A regression model obtained (R^2^ = 0.22) showed how a greater increase in skin perfusion was associated with a higher maximum estimated core temperature during exercise, a higher variation of estimated core temperature at rest and a higher variation of skin temperature.

**Discussion:**

The posterior leg was one of the ROIs with the greatest skin perfusion increase. In conclusion, the anterior thigh can be considered the most appropriate region for evaluating post-exercise skin perfusion responses following aerobic running under warm environmental conditions. These responses are primarily related to the thermal stress generated by the exercise and the environmental conditions.

## Introduction

The study of skin perfusion is of great interest, as it is affected by different vascular pathologies such as peripheral neuropathy, metabolic syndrome, and circulatory failure in hospitalized patients, among others (Girkantaite et al., 2022; Illigens et al., 2013; Morin et al., 2025; Rossi et al., 2006). Additionally, skin perfusion is considered a key outcome for evaluating the effects of interventions on vascular responsiveness and capacity (Florindo et al., 2024; Hou et al., 2021; Mongkolpun et al., 2021, Mongkolpun et al., 2022). In the context of sport and physical activity, measuring skin perfusion in the hours following exercise is important to better understand the prolonged vasodilation and peripheral blood pooling, which reduce venous return and may contribute to post-exercise hypotension and orthostatic intolerance (Halliwill et al., 2013; Seeley et al., 2021). This altered blood flow distribution may also play a role in recovery processes such as glycogen replenishment and plasma volume restoration (Halliwill et al., 2013; Seeley et al., 2021). These applications highlight the value of measuring skin perfusion, however, it is important to recognize that different measurement techniques exist, each with its limitations and advantages.

The two main laser-based imaging techniques used to assess skin perfusion are Laser Speckle Contrast Imaging (LSCI) and Laser Doppler imaging (LDI) (Guven et al., 2023; Nilsson et al., 2025; Sakr, 2010). Although LSCI offers high temporal and spatial resolution, it exhibits several limitations when compared with certain LDI techniques, including greater sensitivity to motion artefacts, assessment of smaller skin surfaces and a narrower sampling depth, as well as increased susceptibility to ambient light interference in certain conditions (Guven et al., 2023; Sakr, 2010). Regarding laser Doppler–based techniques, Laser Doppler flowmetry (LDF) is the most extensively studied method for assessing microcirculatory perfusion, owing to its long-standing use, extensive validation across experimental and clinical settings, and practical feasibility compared with other laser-based imaging modalities (Rajan et al., 2009). LDF operates based on the laser Doppler effect, whereby coherent laser light interacting with moving red blood cells undergoes a frequency shift proportional to their velocity (Nilsson et al., 2025). However, LDF is inherently limited by its small sampling volume (typically <1 mm³) and the requirement for contact probes, which may influence local perfusion and limit spatial representativeness (Gemae et al., 2021). To overcome these limitations, Laser Doppler Perfusion Imaging (LDPI) was developed as an imaging extension of LDF, enabling the assessment of microcirculatory perfusion over larger skin areas using a non-contact scanning approach (Eriksson et al., 2014). In LDPI, a two-dimensional perfusion map is generated by scanning a laser beam across the tissue surface and calculating the Doppler frequency shift of the backscattered light at each measurement point. The resulting pixels are assembled into a color-coded image representing areas of low and high perfusion, thereby improving spatial representativeness and reproducibility (Rajan et al., 2009).

Despite the increasing interest in monitoring skin perfusion during and after exercise, the use of LDPI systems in this context remains limited. A recent study employed LDPI to investigate the potential effect of muscle damage on baseline changes in skin perfusion, although measurements were restricted to the thigh (Carrión-González et al., 2025). While future studies could benefit from using skin perfusion as an internal load marker in athletes such as runners or cyclists, the relatively long scanning times of LDPI systems (Guven et al., 2023) make it more practical to focus on a target region to identify the skin perfusion responses associated with the higher internal load induced by previous training.

The aim of this study was to evaluate the effect of running in moderate intensity domain on skin perfusion five hours after exercise across different regions of interest (ROIs) in the lower limbs. The measurement timepoint of five hours after exercise was selected to investigate prolonged post-exercise responses rather than acute physiological responses to exercise. This decision was based on a previous study that showed that the peak of skin temperature (T_sk_) after running 10 km occurs in this time window (Priego-Quesada et al., 2022). In addition, this study aimed to examine the relationship between these perfusion responses and changes in other physiological and environmental outcomes (T_sk_, heart rate, heart rate variability, estimated core temperature, outdoor environmental temperature, etc.).

## Materials and methods

### Participants

22 participants (17 males and 5 females, **Table 1**) were included in the study. These were divided into two groups: an exercise group (EG), which performed a 50-min run between measurements, and a control group (CG). Both groups were similar in demographic variables (p>0.05, **Table 1**), except for physical activity volume, which the experimental group had higher volumes (p=0.04). This difference arose because participants allocated to the EG were required to be able to comfortably complete the running protocol, whereas some participants assigned to the CG were not regular runners.

**Table 1:** Participants characteristics.

Parameter	Exercise group (EG; N = 11)	Control group (CG; N = 11)
Males - Females	9 - 2	8 - 3
Age (years)	32 ± 12	32 ± 6
Body mass (kg)	75.8 ± 9.0	75.8 ± 18.0
Height (cm)	175 ± 8	177 ± 11
Body fat (%)	19 ± 6	20 ± 8
Physical activity volume (times/week)	5 ± 2	3 ± 2

All values are presented as mean ± SD.

To minimize variability in T_sk_ and skin perfusion, specific instructions were given to the participants. They were asked to refrain from engaging in intense physical exercise the day before and on the day of measurement, except for the activity required by the protocol. Moreover, they were instructed to avoid sunbathing in the last 24 h, as well as drinking coffee, alcohol or other stimulating beverages during the 4 hours before testing. Additionally, heavy meals must be avoided, being the last one consumed at least two hours before data collection. Participants were also asked not to apply creams to their legs and to ensure at least seven hours of sleep the night prior to testing. Verbal confirmation of conformity to these requirements was obtained on testing day.

All participants signed a consent form agreeing to participate, and all procedures established in the Helsinki Declaration were respected. The University’s local ethics committee approved this research.

### Protocol

**Figure 1:**
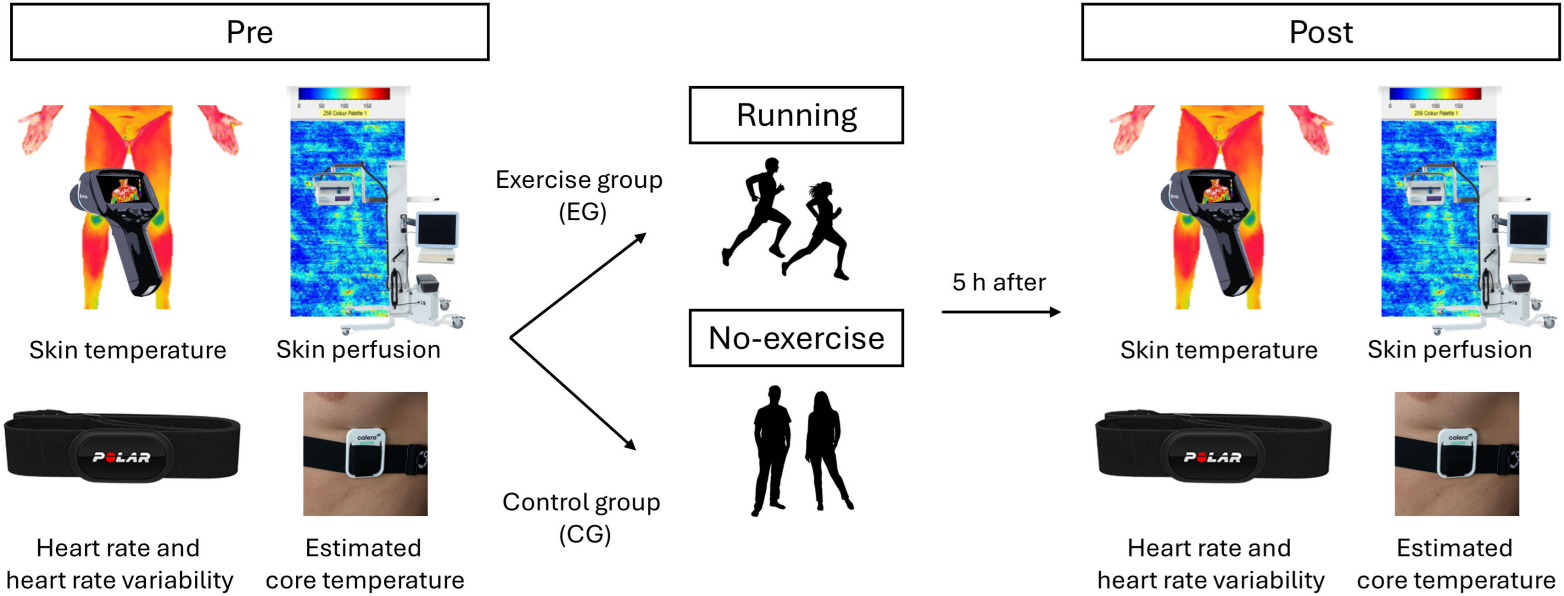
Experimental design illustrating the different phases of the study, comprising one day of testing of two experimental groups (Exercise group vs. Control group).

Data collection was conducted over a single day (**Figure 1**). Participants were first submitted to an anthropometrical assessment with a stadiometer (height) and a bioelectrical impedance system to measure fat percentage (Tanita BC-545M, Tanita Corp., Japan). Then, they completed a 10-min thermal adaptation period in a controlled environment prior to all initial measurements (Marins et al., 2014). Following the adaptation period, infrared thermography measurements were taken under standardized conditions to measure skin temperature (T_sk_). Immediately afterward, a LDPI measurements were performed to assess skin perfusion. Simultaneously, heart rate variability (HRV) was recorded using a chest-worn sensor. Furthermore, during the rest of the protocol, both heart rate (HR) and estimated core temperature (T_c_) were continuously monitored.

After these initial assessments (“Pre” measurement timepoint), participants in the EG completed a 50-min continuous outdoor running session at moderate intensity domain (76 ± 9% of heart rate reserve), while those in the CG were instructed to refrain from any physical activity during the same period of time. Each participant ran in a track with their own running clothing, all of them with shorts and shorts leeved t-shirt. Five hours after the end of the exercise session, all participants returned for a second assessment (“Post” measurement timepoint), during which the same set of measurements was repeated in the same order. All variables were measured under consistent procedures and in the same order for each participant. During the 5-h interval between assessments, participants were allowed to continue their usual daily routines. However, they were instructed to refrain from any structured physical exercise and strenuous physical activity.

It was decided to conduct the study during a period that would facilitate skin perfusion. For this reason, the measurements were taken in Valencia, Spain, in July, in a laboratory with an environmental temperature of 26 °C. All Pre measurements were taken between 8 and 10 am, and all Post measurements between 2 and 4 pm. Environmental conditions were recorded in each session, including room temperature (Pre: 26.2 ± 1.2 °C; Post: 26.1 ± 0.9 °C), indoor relative humidity (Pre: 48 ± 8%; Post: 43 ± 6%), outdoor temperature (Pre: 28.1 ± 1.9 °C; Post: 31.1 ± 2.2 °C), and outdoor relative humidity (Pre: 60 ± 12%; Post: 56 ± 11%). No differences between environmental conditions were observed between groups in any measurement timepoint (p>0.05).

### Measurements

**Figure 2:**
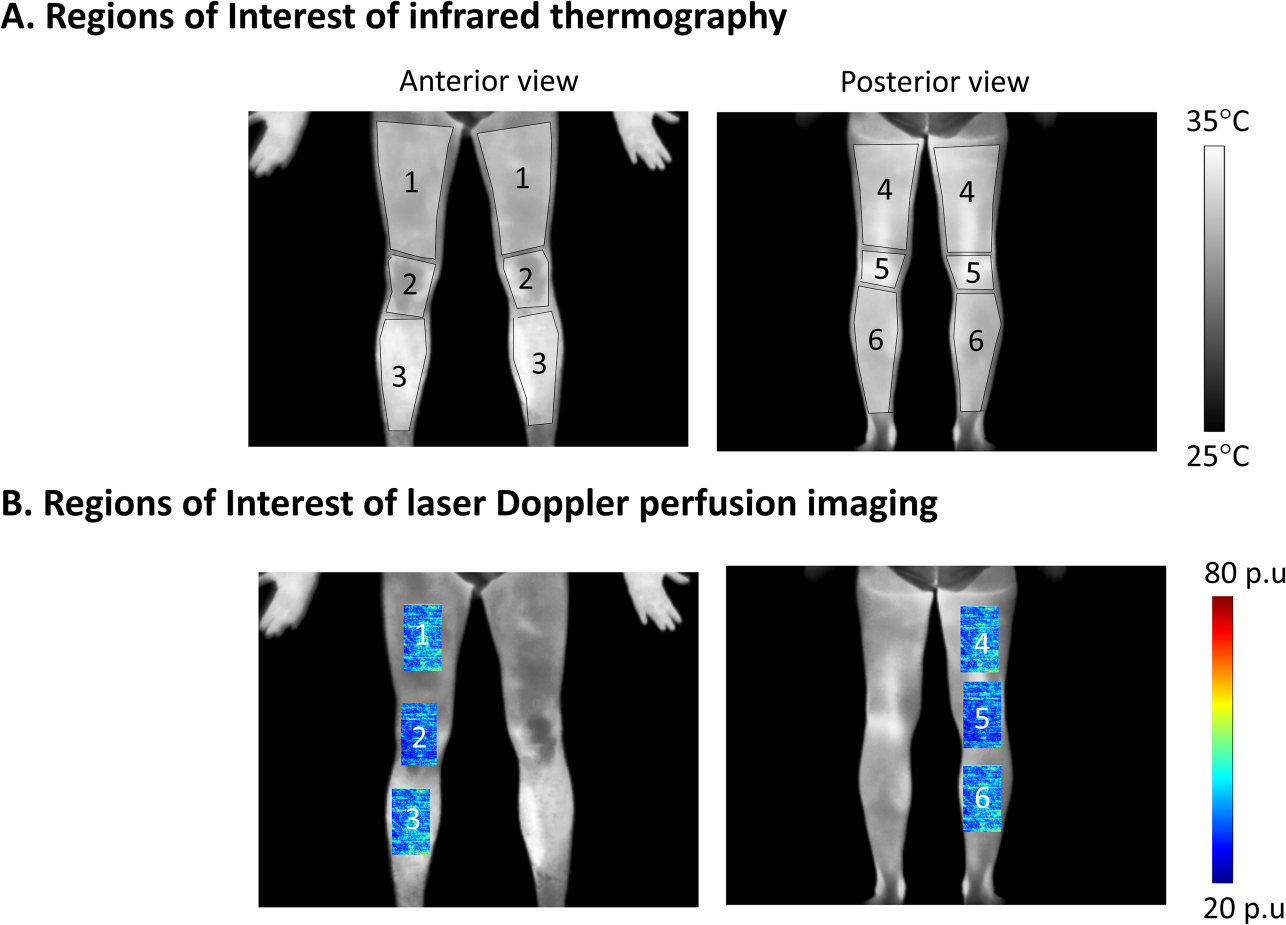
Regions of interest (1-anterior thigh, 2-anterior knee, 3-anterior leg, 4-posterior thigh, 5-posterior knee, and 6-posterior leg) defined to obtain skin temperature **(A)** and skin perfusion **(B)**. Perfusion units, p.u.

T_sk_ was captured using an infrared thermographic (IRT) camera (E54 model, 320 x 240 pixels resolution, an instantaneous field of view (IFOV) of 1.75 mrad, noise-equivalent temperature difference (NETD) <40 mK at 30°, and measurement uncertainty of ± 2 °C or 2% of the reading, Flir Systems Inc., Wilsonville, USA). All IRT procedures followed the Thermographic Imaging in Sports and Exercise Medicine checklist (Moreira et al., 2017). The camera was turned on at least 10-min prior to data collection to ensure optimal thermal stabilization. Before the measurements took place, participants underwent a 10-min adaptation period in an anatomic position (Marins et al., 2014), with both lower limbs exposed (i.e., participants were in underwear with the ROIs uncovered by clothing). The lens of the camera was positioned perpendicular to the ROIs to ensure accuracy in image capture, at a distance of 1 m, and the same evaluator captured all the images. Environmental temperature, relative humidity and reflected temperature (measured according to the standard ISO 18434-1:2008 method) values were entered into the camera software. Six ROIs were defined (**Figure 2A**) on both legs, including the anterior and posterior views of the thigh, knee and lower leg. Thermal data were analyzed using the ThermaCAM Researcher Pro software (version 2.10, FLIR, Wilsonville, USA) to obtain the mean T_sk_ from each ROI, using an emissivity of 0.98 (Steketee, 1973).

A LDPI device (moorLDI2, Moor Instruments Ltd., Devon, United Kingdom) was used to measure skin perfusion. Scans were performed in the supine and prone positions to assess the anterior and posterior ROIs of the lower leg, respectively. Being the first scan 3-min after being in that position, and maintaining a laser-leg distance of 45 cm. To minimize scanning time and reduce motion artefacts, the image size was restricted to a small area covering each ROI. Scans were acquired at a resolution of 150 × 207 pixels (7.7 × 10.5 cm) and a scanning speed of 4 ms/pixel, with a total acquisition time of 2-min and 43-sec. Six ROIs (**Figure 2B**, same body segments as for the T_sk_) were measured in the preferred lower limb, always in the same order (anterior thigh, anterior knee, anterior leg, posterior thigh, posterior knee and posterior leg). Lower limb preference was determined by asking the question: *“If you would kick a ball on a target, which leg would you use to kick the ball?”* (van Melick et al., 2017). The six ROIs were defined by marking, with a permanent marker, the central point and corners of each segment to allow consistent post-timepoint measurements. To minimize distortions in the images, participants were instructed to remain still during scans. Mean skin perfusion in perfusion units (p.u.) was obtained using the manufacturer’s software (Moor LDI Review Software, version 6.2; Moor Instruments Ltd., Devon, UK).

In addition, Δskin perfusion and Δskin temperature were obtained as the difference between Post and Pre timepoints, in a percentage and absolute value, respectively.

HR and HRV data were measured using a chest strap (H10, Polar Electro Oy, Finland) connected via Bluetooth to the Elite HRV mobile application (version 5.5.12), which enabled real-time data acquisition. The recording lasted a total time of 3-min in the lying supine position. During that time, participants were instructed to remain silent, with closed eyes, still and breathe spontaneously throughout the assessment. Data was exported from the application, and processing was performed using the “RHRV” package (version 5.0.0) (Martinez et al., 2017) in RStudio (version 2025.09.1). After data filtering using the package’s automatic and manual methods, the time domain (using a window size of 60-sec) and frequency domain (using the Fourier Transform) of HRV was performed. Variables extracted were the Standard Deviation of the NN interval (SDNN), the mean of the Standard Deviation calculated over the windowed intervals (SDNN index), the Root Mean Square of Successive Differences (RMSSD), and the High Frequency component (HF). The percentage variation concerning the Pre measurement timepoint was assessed (Laborde et al., 2017). In the case of the HR, the variation was obtained in absolute values and not in percentages.

Estimate T_c_ was monitored throughout the entire protocol using a heat flux sensor (Calera Research™ sensor, firmware version 0.8.6, GreenTeg, Rümlang, Switzerland) synchronized with a Garmin watch (Forerunner 55, Garmin International, Inc., Kansas, USA). The device was attached to the chest strap located on the left side of the HR monitor band (close to the armpit). The device recorded data every second and was paired with the HR monitor to improve the data accuracy as recommended by the manufacturer. Data during the lying supine and during exercise (and corresponding time for the CG) were obtained. During exercise, mean and maximum T_c_ were obtained. ΔT_c_ was obtained as the difference between Post and Pre timepoints.

### Statistical analysis

Statistical analyses were performed using RStudio (version 2025.09.1) and an alpha set at 0.05. Data are reported with mean, standard deviation, and 95% confidence interval (95%CI) of the differences between conditions. The distribution of each variable was assessed using the Shapiro–Wilk test and visual inspection of the data distributions. Parametric tests were applied when the assumptions of normality were reasonably met, whereas non-parametric alternatives were used for variables showing clear departures from normality. The main effect of leg preference was not significant in the baseline T_sk_ ANOVA (p=0.68), nor was its interaction with the other factors (p>0.59). Therefore, this factor was not considered in the analysis of the results. For the analysis of the baseline data (Pre), differences between groups (CG vs. EG) were assessed for absolute values using Student T-tests for parametric data, and Wilcoxon tests for non-parametric data (anterior and posterior legs of skin perfusion, anterior leg, posterior thigh, posterior knee and posterior leg of T_sk_). For Δ values of the different variables, Student-T tests were performed to assess the differences between groups for parametric data (mean and maximum values of T_c_ during exercise) and Mann-Whitney U tests for non-parametric data (HRV variables and ΔT_c_). For the Δ values of skin perfusion and T_sk_ which includes two factors (measurement timepoint and ROI), the Friedman test with Mann-Whitney U *post-hoc*s (for group differences) and Wilcoxon *post-hoc*s (for ROI differences) were employed. Bonferroni correction was applied for all the comparisons. For significant pair differences, Hedge’s effect sizes (ES) were computed and classified as small (ES 0.2–0.5), moderate (ES 0.5–0.8), or large (ES>0.8) (Cohen, 1988; Hedges, 1981). An exploratory analysis based on a multiple regression analysis was performed to assess the influence of different factors on Δskin perfusion. The inputs introduced in the regression were: ROI, ΔT_sk_ (mean of both lower limbs), ΔHR, ΔRMSSD, ΔHF, ΔT_c_ at rest, maximum T_c_ during running, ΔHF, and body fat. In addition, the relative contribution of each predictor to the model was quantified by calculating the proportion of variance explained by each factor. For this purpose, the sum of squares associated with each term in the regression model was divided by the total model sum of squares, providing an estimate of the percentage of variance uniquely accounted for by each predictor. This metric facilitated the comparison of the relative explanatory weight of the different physiological and environmental variables included in the model. Finally, to compare with previous literature, the relationship between absolute values of T_sk_ and skin perfusion was analyzed using a linear simple regression considering all the data (all the ROIs and both measurement timepoints).

## Results

### Skin perfusion and skin temperature

Because the statistical analysis has been carried out with delta values, **Table 2** shows the values at the Pre measurement timepoint to describe the starting point of each group. Skin perfusion of most of the ROIs presented similar values at Pre (p>0.20), except the anterior thigh, which had higher values for the CG (95%CI of the difference [3, 44 a.u.], p=0.02 and ES = 1.0). Any of the ROIs presented T_sk_ differences between the CG and EG at Pre timepoint (p<0.23).

**Table 2:** Mean ± standard deviation of skin perfusion and skin temperature at the Pre measurement timepoint.

Skin perfusion (p.u)	EG	CG	P-value
Anterior thigh	81 ± 18	104 ± 26	**0.02**
Anterior knee	71 ± 17	70 ± 17	0.93
Anterior leg	74 ± 16	81 ± 27	1.00
Posterior thigh	69 ± 14	76 ± 16	0.22
Posterior knee	78 ± 14	90 ± 27	0.20
Posterior leg	68 ± 11	78 ± 25	0.52
Skin temperature (°C)			
Anterior thigh	31.2 ± 1.0	31.0 ± 1.0	0.49
Anterior knee	30.5 ± 0.9	30.5 ± 1.0	0.89
Anterior leg	31.7 ± 0.6	31.6 ± 0.8	0.82
Posterior thigh	31.5 ± 0.7	31.4 ± 1.0	0.64
Posterior knee	31.9 ± 0.5	31.7 ± 0.9	0.97
Posterior leg	31.7 ± 0.5	31.4 ± 0.8	0.23

Significant values are indicated in bold letters.

P-value was obtained between the Experimental and Control group (EG vs. CG). p.u: perfusion units.

**Figure 3:**
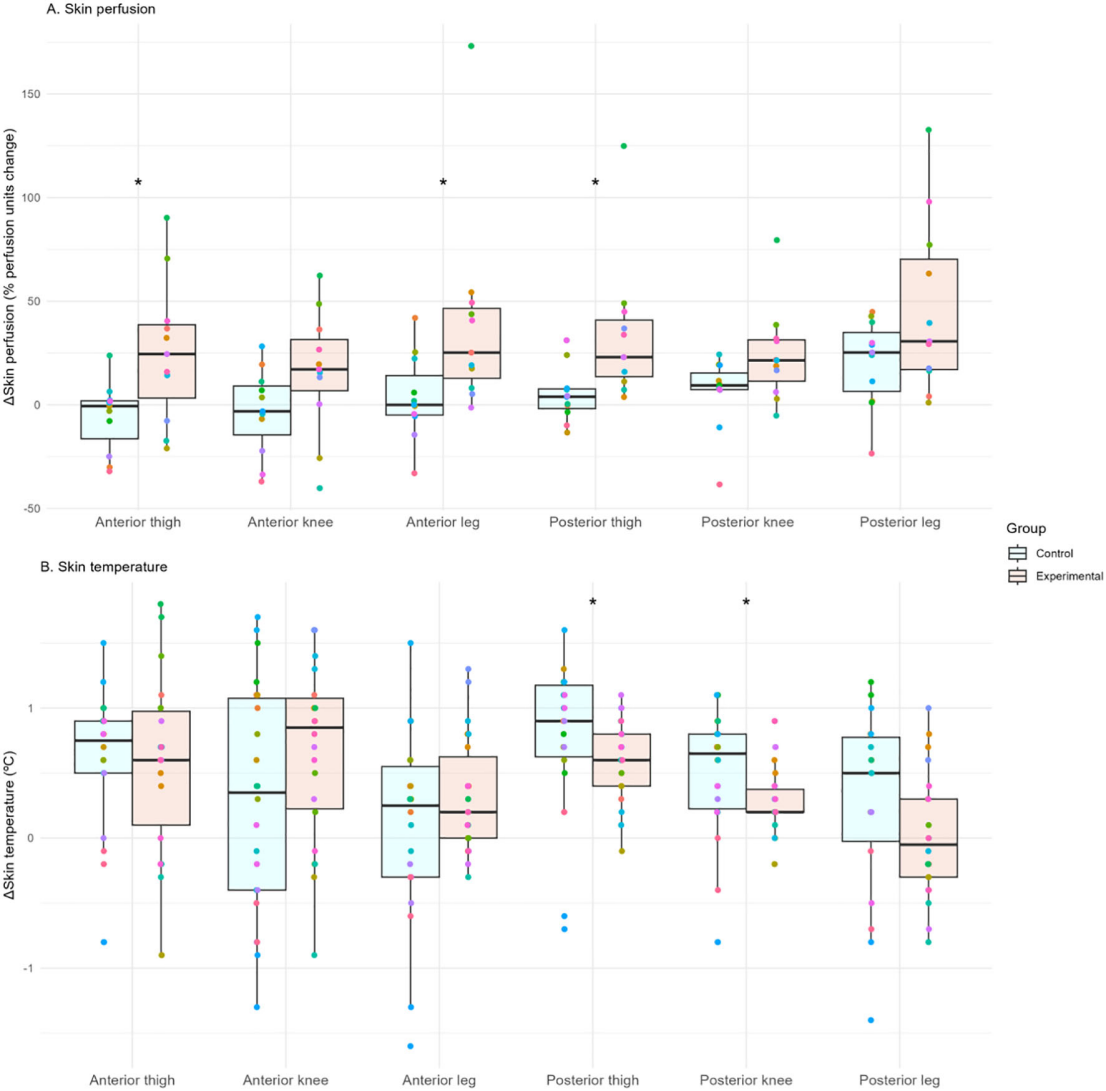
Box plots (median, interquartile range, and whiskers to 1.5× interquartile range) of the skin perfusion **(A)** and skin temperature **(B)** variation (difference between post and pre) of the control and experimental groups. Differences between groups are indicated using * (p<0.05). Points indicated the individual values.

**Figure 3A** shows the Δskin perfusion of the two groups assessed. The EG presented a higher increase of skin perfusion than the CG for the anterior thigh (95%CI[7, 58%], p=0.02 and ES = 1.0), anterior leg (95%CI[1, 79%], p=0.04 and ES = 0.3), and posterior thigh (95%CI[8, 56%], p<0.01 and ES = 0.5). Non-differences were observed between ROIs on skin perfusion of Δ values for the EG (p>0.28). For the CG, the posterior leg presented a higher skin perfusion increase than the anterior thigh (95%CI[10, 43%], p=0.02 and ES = 1.0).

**Figure 3B** shows the ΔT_sk_ of the two groups assessed. The EG presented a lower increase of T_sk_ at Post than the CG for the posterior thigh (95%CI[0.0, 0.5 °C], p=0.04 and ES = 0.4), and posterior knee (95%CI[0.0, 0.5 °C], p=0.02 and ES = 0.4). The EG had lower increases of T_sk_ for the posterior leg than the anterior knee (95%CI[0.4, 1.0 °C], p<0.01 and ES = 1.1), the anterior leg (95%CI[0.2, 0.5 °C], p=0.02 and ES = 0.7), and the posterior thigh (95%CI[0.4, 0.9 °C], p=0.01 and ES = 1.4). The posterior knee also presented lower increases than the posterior thigh (95%CI[0.2, 0.5 °C], p<0.01 and ES = 1.1). Considering the CG, the anterior leg had lower increases than the anterior thigh (95%CI[0.3, 0.6 °C], p<0.01 and ES = 0.7), the posterior thigh (95%CI[0.5, 1.0 °C], p<0.01 and ES = 1.0), and the posterior knee (95%CI[0.3, 0.6 °C], p=0.01 and ES = 0.6). The posterior thigh also had, for the CG, higher increases than the posterior knee (95%CI[0.2, 0.5 °C], p=0.01 and ES = 0.5) and posterior leg (95%CI[0.3, 0.7 °C], p<0.01 and ES = 0.8).

### Core temperature and heart rate

The EG presented at Pre a HR of 57 ± 9 beats/min and the CG a HR of 60 ± 8 beats/min. The HRV variables did not present differences between groups in their variation between both measurement timepoints (**Table 3**; p>0.13).

**Table 3:** Mean ± standard deviation of variation of heart rate (HR), heart rate variability (HRV) variables (SDNN - standard deviation of the NN interval, SDNN index - mean of the standard deviation calculated over the windowed intervals, RMSSD - root mean square of successive differences, and HF - high frequency component) and estimated core temperature.

ΔHRV variables	EG	CG	P-value
HR (beats/min)	2 ± 6	-1 ± 4	0.13
SDNN (%)	-4 ± 20	5 ± 20	0.14
SDNNIDX (%)	1 ± 21	3 ± 24	0.98
RMSSD (%)	-4 ± 22	7 ± 22	0.49
HF (%)	-11 ± 27	15 ± 73	0.26
Estimated core temperature (°C)			
ΔT_c_ at Rest (Post-Pre; °C)	-0.2 ± 0.9	0.1 ± 0.3	0.29
ΔT_c_ at Exercise (Max exercise-Pre; °C)	1.5 ± 0.7	0.2 ± 0.3	**<0.001**

Significant p-values are highlighted in bold letters. P-value was obtained between the Experimental and Control group (EG vs. CG).

Regarding T_c_ (**Table 3**), the EG had at Pre 37.3 ± 0.9 °C and the CG 36.9 ± 0.3 °C, and during exercised EG reached values of 38.8 ± 0.6 °C and the CG without performing exercise of 37.1 ± 0.2 °C Both groups did not differ in their ΔT_c_ variation at rest (p=0.29), but during the exercise time the EG presented higher T_c_ values (mean: 95%CI[0.8, 1.8 °C], p<0.001 and ES = 1.1; max: 95%CI[0.2, 1.1 °C], p<0.01 and ES = 4.0).

### Regression analysis

The regression model obtained explained 22% of the variance of the Δskin perfusion (**Table 4**). In order of percentage of variance explained, a greater increase in skin perfusion was associated with a higher maximum T_c_ during exercise, a higher ΔT_sk_, and a higher ΔT_c_ at rest. Furthermore, the posterior leg was one of the ROIs with the greatest skin perfusion increase. Δ in HRV and body fat were not related to this increase in skin perfusion (p>0.2).

**Table 4:** Multivariate regression model obtained explaining variation (Post – Pre) in skin perfusion.

Variable	Coefficient	95%CI	P-value	Explained variance (%)
**(Intercept)**	-811	-1,304, -318	**<0.01**	
**ROI**			**0.03**	9.7
*Anterior thigh*	—	—		
*Knee anterior*	-6.7	-26, 12	0.5	
*Leg anterior*	12	-6.7, 32	0.2	
*Posterior thigh*	6.8	-12, 26	0.5	
*Posterior knee*	6.1	-13, 25	0.5	
*Posterior leg*	29	10, 49	**<0.01**	
**ΔT_sk_ (°C)**	10	1.5, 21	**0.02**	3.5
**ΔHR (beats/min)**	0.62	-1.2, 2.5	0.5	4.7
**ΔRMSSD (%)**	0.09	-0.44, 0.63	0.7	2.5
**ΔHF (%)**	-0.01	-0.14, 0.13	0.9	1.3
**ΔT_c_ at rest (°C)**	16	4.5, 27	**<0.01**	2.3
**Maximum T_c_ during exercise (°C)**	15	3.9, 25	**<0.01**	6.8
**Body fat (%)**	0.27	-0.91, 1.4	0.7	0.1
*R²*	0.31			
*Adjusted R²*	0.22			
*Overall p-value of the model*	<0.001			

Significant p-values are highlighted in bold letters. Inputs of the model were the region of interest measured (ROI), variations of skin temperature in which ROI (ΔT_sk_), heart rate (ΔHR), root mean square of successive differences of heart rate variability (ΔRMSSD), high frequency component of heart rate variability (ΔHF), core temperature (ΔT_c_), maximum T_c_ during running, and body fat.

Absolute values of T_sk_ and skin perfusion showed a direct relationship between them (intercept -322 ± 46; T_sk_ coefficient 12.9 ± 1.5 p<0.001 and R^2^ = 0.23).

## Discussion

This study aimed to evaluate the effect of 50-min running in a moderate intensity domain on skin perfusion five hours after exercise across different ROIs in the lower limbs. Additionally, the relationship between changes in skin perfusion and variations in other physiological and environmental variables was explored. The main results were that the EG showed greater increases in skin perfusion than the CG at the anterior thigh (large ES), anterior leg (small ES) and posterior thigh (moderate ES). Furthermore, greater increases in skin perfusion variation were associated with higher T_c_ during exercise, a higher ΔTsk, and a higher ΔTc at rest.

Skin perfusion can be a valuable internal load variable to monitor in athletes (Florindo et al., 2024; Halliwill et al., 2013; Seeley et al., 2021). In this regard, the use of LDPI has associated advantages (Eriksson et al., 2014), although one of its main limitations is the relatively long scanning time required for each ROI (Guven et al., 2023), which may constrain its use when measurement time overlaps with other physiological or biomechanical assessments. Consequently, understanding the regional behavior of skin perfusion after exercise is essential to prioritize specific anatomical sites and optimize data collection protocols. The present findings suggest that the anterior thigh represents a primary area of interest five hours after a 50-min running bout, as it exhibited the greatest differentiation compared with non-exercising controls. Secondary regions of interest included the anterior leg and posterior thigh, although with smaller effect sizes (0.3-0.5). The pronounced response observed at the anterior thigh may be explained by the high activation of the large muscle groups located in this segment during running, particularly the quadriceps femoris, which displays substantial recruitment across a wide range of running intensities (Sloniger et al., 1997). Elevated muscle activation is associated with increased metabolic heat production (Fletcher and MacIntosh, 2017), leading to greater thermoregulatory demands and sustained cutaneous vasodilation to facilitate heat dissipation (Charkoudian, 2003; Cramer and Jay, 2016). It is important to note that the skin perfusion was assessed five hours after exercise cessation, indicating that the observed elevations are unlikely to be solely explained by acute thermoregulatory responses. Instead, prolonged post-exercise mechanisms may be involved, including sustained endothelial-dependent vasodilation, residual metabolic activity, local inflammatory processes, and exercise-induced increases in nitric oxide bioavailability, all of which have been shown to enhance cutaneous microvascular function beyond the immediate recovery period (Hellsten and Nyberg, 2016; Kvernmo et al., 1998; Wang, 2005). Finally, it should be noted that all measurements were performed under warm environmental conditions, which likely favored peripheral vasodilation and facilitated the detection of regional differences in skin perfusion (Charkoudian, 2003). Under cooler conditions, enhanced sympathetic vasoconstrictor activity might attenuate post-exercise skin blood flow responses, potentially reducing regional discrimination several hours after exercise (Charkoudian, 2003).

The posterior leg was the ROI that exhibited the greatest increases in skin perfusion, with no differences between the EG and the CG. This finding was consistent with the results of the multiple regression analysis and with the greater perfusion changes observed in this region compared with the anterior thigh in the control group. Several mechanisms may account for this response. First, circadian rhythms exert a significant influence on skin blood flow, with progressively increasing perfusion throughout the day (Smolander et al., 1993). Previous studies have shown that distal skin sites exhibit greater circadian-related fluctuations in T_sk_ compared with more proximal regions, reflecting their role in heat dissipation and thermoregulatory control (Costa et al., 2015; Zaproudina et al., 2008). In addition, the posterior region of the leg contains the main neurovascular pathways supplying the distal lower limb, including the popliteal vessels and their bifurcation into the posterior tibial and fibular arteries, which course deeply through the posterior compartment. This anatomical arrangement may contribute to a higher baseline perfusion and a greater susceptibility to systemic or centrally mediated increases in skin blood flow, independently of local muscular activity. Beyond circadian and anatomical factors, previous work has also highlighted a coupling between skin perfusion, blood temperature, and intravascular ATP availability, which may contribute to vasodilation in both active and non-active tissues during and after exercise (González-Alonso et al., 2015). From a practical perspective, these findings suggest that the posterior leg may be a suitable site for assessing general peripheral vascularization status, but it appears less sensitive for detecting exercise-induced changes in skin perfusion.

The multiple regression analysis provides insight into the main factors explaining the variance in post-exercise increases in skin perfusion. T_c_ during exercise emerged as a key differentiating variable between groups, reflecting the magnitude of thermal stress induced by the running bout. This finding is physiologically coherent, as higher T_c_ increases the demand for heat dissipation, primarily through elevations in skin perfusion (Charkoudian, 2003; Cramer and Jay, 2016). Accordingly, T_c_ appears to be a central integrative variable linking exercise intensity, metabolic heat production, and subsequent thermoregulatory responses (Cramer and Jay, 2016; Kenny and Journeay, 2010). This is also coherent with the effect observed of ΔT_c_ at rest with skin perfusion. These findings should be interpreted in light of the environmental context of the intervention, which was conducted during a warm season and is therefore biased toward conditions that favor peripheral vasodilation. Future studies should investigate post-exercise skin perfusion responses across different seasons and environmental conditions, as well as under varying internal load states, to better delineate the independent and interactive effects of thermal stress, and exercise intensity on cutaneous vascular responses.

Several studies have reported a strong association between T_sk_ and skin perfusion, as increases in cutaneous blood flow elevate T_sk_ through convective heat transfer, whereas vasoconstriction is typically accompanied by reductions in T_sk_ (Carrión-González et al., 2025; Schlager et al., 2010). This is also supported by our results, which show that the 22% of the variance of the skin perfusion is explained by the T_sk_. Higher variances of other studies could be explained as they produce higher differences in both outcomes by cold stress protocols or by including patients with skin perfusion pathologies (Murray et al., 2009; Schlager et al., 2010). The novelty of our results is to show that also Δskin perfusion produced by exercise is related to ΔT_sk_, but only explains a 2% of the variance. This apparent dissociation may be explained by the multifactorial regulation of T_sk_ (Fernández-Cuevas et al., 2015). While skin perfusion is closely linked to internally generated thermal stress, particularly in warm environments, T_sk_ is also strongly influenced by evaporative heat loss through sweating and by environmental conditions, which may obscure its relationship with microvascular blood flow (Priego Quesada et al., 2016; Xu et al., 2013). Similarly, neither HR nor HRV indices were associated with skin perfusion. Although cardiac autonomic modulation influences peripheral vascular tone, HRV primarily reflects cardiac parasympathetic activity and global autonomic balance rather than regional vasomotor control (Michael et al., 2017). In this context, vagally mediated HRV indices such as HF or RMSSD are sensitive to exercise intensity, recovery kinetics, respiration, and methodological factors, but may lack the spatial specificity required to capture localized or delayed cutaneous microvascular responses (Laborde et al., 2017). Furthermore, HRV derived metrics are known to exhibit reduced sensitivity during and following exercise, particularly when variability is markedly suppressed and signal-to-noise ratio is diminished (Gronwald and Hoos, 2020). As highlighted in methodological and applied reviews, HRV should therefore be interpreted as an integrative marker of cardiac autonomic regulation rather than a direct surrogate of peripheral vascular function (Laborde et al., 2017; Mosley and Laborde, 2024). This distinction may explain the absence of associations between HRV indices and skin perfusion in the present study, especially given that perfusion was assessed several hours after exercise cessation.

In addition to the limitations already mentioned (e.g., results associated with heat conditions), another important limitation of this study is its restriction to only 5 hours post-exercise. This was done to recruit a sufficient sample within one month and avoid extending the experimental phase further, which could lead to changes in external environmental conditions. Nevertheless, given the novelty of this study, it can be considered a good starting point for future research. Another limitation was that the exercise group reported a higher physical activity volume than the control group. This difference resulted from the requirement that participants assigned to the exercise group were able to complete the 50-min running protocol. Although physical activity volume was not identified as a significant predictor of changes in skin perfusion, future studies should aim to match groups more closely for training status to further isolate the effects of the exercise intervention. Finally, anterior thigh skin perfusion differed between groups at baseline. No differences in environmental conditions or body fat percentage were identified that could explain this imbalance. Because the primary analyses were based on changes from baseline, the impact of absolute baseline differences on the main findings was minimized. Given the absence of an identifiable explanatory factor, this difference likely reflects the high inter-individual variability characteristic of skin perfusion measurements.

## Conclusions

The anterior thigh appears to be the most appropriate region for assessing post-exercise skin perfusion responses following aerobic running under warm environmental conditions. These responses seem to be primarily driven by the thermal stress generated during exercise and may be enhanced by the substantial activation of the quadriceps femoris, together with the influence of environmental temperature.

## Data Availability

The raw data supporting the conclusions of this article will be made available by the authors, without undue reservation.
